# An integrative genomic approach reveals coordinated expression of intronic miR-335, miR-342, and miR-561 with deregulated host genes in multiple myeloma

**DOI:** 10.1186/1755-8794-1-37

**Published:** 2008-08-13

**Authors:** Domenica Ronchetti, Marta Lionetti, Laura Mosca, Luca Agnelli, Adrian Andronache, Sonia Fabris, Giorgio Lambertenghi Deliliers, Antonino Neri

**Affiliations:** 1 Department of Medical Sciences, Leukemia Study Center, University of Milan, Italy; 2 Hematology 1 Fondazione IRCCS Policlinico, Milan, Italy

## Abstract

**Background:**

The role of microRNAs (miRNAs) in multiple myeloma (MM) has yet to be fully elucidated. To identify miRNAs that are potentially deregulated in MM, we investigated those mapping within transcription units, based on evidence that intronic miRNAs are frequently coexpressed with their host genes. To this end, we monitored host transcript expression values in a panel of 20 human MM cell lines (HMCLs) and focused on transcripts whose expression varied significantly across the dataset.

**Methods:**

miRNA expression was quantified by Quantitative Real-Time PCR. Gene expression and genome profiling data were generated on Affymetrix oligonucleotide microarrays. Significant Analysis of Microarrays algorithm was used to investigate differentially expressed transcripts. Conventional statistics were used to test correlations for significance. Public libraries were queried to predict putative miRNA targets.

**Results:**

We identified transcripts specific to six miRNA host genes (*CCPG1*, *GULP1*, *EVL*, *TACSTD1*, *MEST*, and *TNIK*) whose average changes in expression varied at least 2-fold from the mean of the examined dataset. We evaluated the expression levels of the corresponding intronic miRNAs and identified a significant correlation between the expression levels of *MEST*, *EVL*, and *GULP1 *and those of the corresponding miRNAs miR-335, miR-342-3p, and miR-561, respectively. Genome-wide profiling of the 20 HMCLs indicated that the increased expression of the three host genes and their corresponding intronic miRNAs was not correlated with local copy number variations. Notably, miRNAs and their host genes were overexpressed in a fraction of primary tumors with respect to normal plasma cells; however, this finding was not correlated with known molecular myeloma groups. The predicted putative miRNA targets and the transcriptional profiles associated with the primary tumors suggest that *MEST*/miR-335 and *EVL/*miR-342-3p may play a role in plasma cell homing and/or interactions with the bone marrow microenvironment.

**Conclusion:**

Our data support the idea that intronic miRNAs and their host genes are regulated dependently, and may contribute to the understanding of their biological roles in cancer. To our knowledge, this is the first evidence of deregulated miRNA expression in MM, providing insights that may lead to the identification of new biomarkers and altered molecular pathways of the disease.

## Background

Multiple myeloma (MM) is a plasma cell neoplasia characterized by profound genomic instability involving numerical and structural chromosomal aberrations [1]. The availability of human MM cell lines (HMCLs) has been of critical importance in revealing many of the molecular and biological aspects of MM. Over the last few years, recurrent nonrandom genetic lesions have been identified that seem to correlate with the clinical course of MM and its response to therapy. Nearly half of MM tumors are nonhyperdiploid, and frequently show chromosome 13 deletion and constitutively activated *CCND1 *(11q13), *CCND3 *(6p21), *MAF *(16q24), *MAFB *(20q12), or *FGFR3/MMSET *(4p16.3) as a result of chromosomal translocations involving the immunoglobulin heavy chain locus (*IGH@*) on chromosome 14q32 [2-5]. The remaining tumors are hyperdiploid, which are characterized by multiple trisomies of nonrandom odd chromosomes, and a low prevalence of *IGH *translocations and chromosome 13 deletions [1].

The recent discovery of microRNA (miRNA) genes encoding a class of small (17–25 base pairs) noncoding RNAs involved in the regulation of the cell cycle, survival, and differentiation programmes has added a further level of complexity to normal and cancer cell biology. Through complementary base pairing to specific protein-coding mRNA transcripts, miRNAs direct mRNA silencing by different mechanisms, including message degradation and translational repression [6]. Several studies have reported that chromosomal abnormalities and/or epigenetic events contribute to miRNA deregulation; impaired miRNA expression has already been demonstrated in a number of solid tumors and, more recently, in some hematological disorders [7-9]. To date, miRNA expression and deregulation in MM remain to be investigated; recently, it has been demonstrated that miR-21 can be induced by STAT3 and mediate IL-6-dependent HMCL survival [10].

Approximately one third of miRNAs are located within the intronic regions of coding transcription units [11-13]. The expression of these miRNAs largely coincides with the transcription of the corresponding host genes, which suggests that they can share the same regulatory sequences as their host transcription units [11] and can be cotranscribed with them under the regulation of the RNA polymerase II (PolII) following the coordinated processing of intronic miRNAs and cognate mRNA [14]. However, the mechanism of intronic miRNA maturation remains to be fully understood, because miRNAs in an antisense orientation to their corresponding host gene may possess independent regulatory motifs [12], and miRNAs located within genomic repetitive elements may be transcribed by RNA polymerase III (PolIII) [15].

In the present study, we investigated the expression of miRNAs located within transcription units found to be differentially expressed in a panel of HMCLs that we recently profiled using gene expression microarrays [16]. This approach led to the identification of three miRNAs (miR-335, miR-342-3p, and miR-561) that were differentially expressed, along with their corresponding host genes, in the dataset of HMCLs. In addition, we found that overexpression of these miRNAs/host genes was recurrent in MM primary tumors compared with normal plasma cells. The data discussed here may suggest a possible role for deregulated intronic miRNA species in myeloma.

## Methods

### Cell lines

The HMCLs were obtained from DMSZ-German collection of Microorganisms and Cell Culture, Germany (NCI-H929, OPM2, JJN3, RPMI-8226, and KMS-12); or kindly provided by Dr. T. Otsuki, Kawasaki Medical School, Okayama, Japan (KMS-28, KMS-34, KMS-18, KMS-11, KMS-26, KMS-27, KMM-1 and KMS-20); Dr S. Iida, Nagoya City University Graduate School of Medical Sciences, Nagoya, Japan (KM4, FR4, and AMO1), and Dr. F. Malavasi, Department of Genetics, University of Torino, Italy (LP-1); or were established in our laboratory (CMA-01, CMA-02 and CMA-03) [17]. They were cultured in Iscove's modified Dulbecco's medium supplemented with 10% fetal calf serum at concentrations ranging from 3 × 10^5 ^to 8 × 10^5^, at 37°C in a 5% CO_2 _humidified atmosphere. CMA-01, CMA-02 and CMA-03 were cultured in presence of 20 U/ml recombinant human IL-6 (R&D System, Minneapolis, MN, USA).

### Specific miRNA quantification by RealTime RT-PCR

Total RNA was extracted from at least 2 × 10^6 ^purified plasma cells by using Trizol reagent. Quantitative assessment of the RNA was performed using Nanodrop ND-1000 Biophotometer (NanoDrop Technologies): the minimum OD_260/280 _ratio to be considered acceptable is 1.98–2.10. In the reverse transcription step, 50 ng total RNA was employed in RT reactions using reagents from the TaqMan^R ^MicroRNA RT kit (Applied Biosystems) and specific miRNA primers provided with the TaqMan^R ^MicroRNA Assays. 15 μl reactions were incubated in an Applied Biosystems 9700 Thermocycler. All reverse transcriptase reactions were run in duplicate. Real Time PCR was performed in triplicate using TaqMan^R ^MicroRNA Assays together with the TaqMan^R ^Universal PCR Master Mix on an Applied Biosystems 7700 Sequence Detection System. All RNA samples were normalized based on the *Z30 *TaqMan^R ^MicroRNA Assays-Control. The threshold cycle (C_T_) was defined as the fractional cycle number at which the fluorescence passes the fixed threshold. All signals with C_T _≥ 40 were manually set to undetermined. Relative quantification of miRNA expression was calculated with the 2^-ΔCt ^method (Applied Biosystem User Bulletin N°2). Data were presented as relative quantity of target miRNA, normalized to *Z30 *housekeeping gene.

### Bioinformatic Analysis

mRNA targets were predicted for the 3 miRNA of interest by querying four different bioinformatic algorithms which are miRanda [18,19], TargetScan [20,21], PicTar [22,23], and Diana microT [24,25].

### Genome-wide DNA profiling analysis

Genome-wide DNA profiling was performed on Affymetrix GeneChip Human Mapping 250 k NspI arrays. To find the corresponding copy number (CN) values, we firstly extracted the raw data from the CEL files using the Affymetrix packages GTYPE 4.1 and Copy Number Analysis Tool 4.0.1 (Affymetrix, Santa Clara, CA, USA) and the Mapping Array 250 k NspI probe annotations released on July, 12 2007. In order to keep only the raw data and thus to avoid the CN inference facility of the latter software package, the Hidden Markov Model Genomic Smoothing window was set to 0. After the preprocessing, piecewise constant estimates of the underlying local DNA CN variation was calculated using the DNA copy Bioconductor package, which looks for optimal breakpoints using circular binary segmentation (CBS). In order to overcome scaling biases related to the greatly altered ploidy of HMCLs (reflected in a median value for SNP probes different from two in almost all samples) the median of the estimated profiles for each sample was scaled back to assign to a nominal multiplicity of two those values of probes mapped to regions for which FISH information was available and indicated the presence of exactly two alleles [16]. After scaling, a k-means clustering algorithm was used to determine the interval values for inferring discrete CN values. As such, inferred CN higher than 1.73, 2.16, and 2.64 corresponded to two, three or more than four DNA copies, respectively; whereas CN below 1.73, and 1.37 to one copy or biallelic deletion, respectively. CN data of these HMCLs have been deposited in National Center for Biotechnology Information's Gene Expression Omnibus (GEO; ) and are accessible through GEO Series accession number GSE11522 [26].

### Gene expression profiling database

Patients database included the proprietary database (4 normal samples, 12 MGUS, 132 MM, and 9 PCL), together with 20 normal samples, 22 MGUS, and 137 MM taken from 2 publicly available MM gene expression datasets [1,27] (GSE6477 and GSE6691, CEL files available at Gene Expression Omnibus [26]), all profiled on HG-U133A. The probe level data were converted to expression values using the Bioconductor function for the Robust Multi-Array average (RMA) procedure [28], and the absence of outlier patients in the normalization process due to hybridization signals was verified by Expression Console tools (Affymetrix, Santa Clara, CA, USA). The supervised analyses were performed using SAM software version 3.0 [29,30]. The cut-off for significance was determined by tuning the Delta parameter on the false discovery rate (FDR) and controlling the q-value for the gene list (at a q-value = 0). The selected lists were functionally analyzed using the Database for Annotation, Visualization and Integrated Discovery (DAVID) Tool 2006 (U.S. National Institutes of Health [31]) and NetAffx [32].

## Results

### Identification of intronic miRNAs deregulated in HMCLs

We searched for potentially deregulated miRNAs mapping to the intronic, exonic, or 3' UTR regions of the most differentially expressed transcripts in a proprietary dataset of 20 HMCLs previously profiled on HG-U133A GeneChip arrays [16].

**Figure 1:**
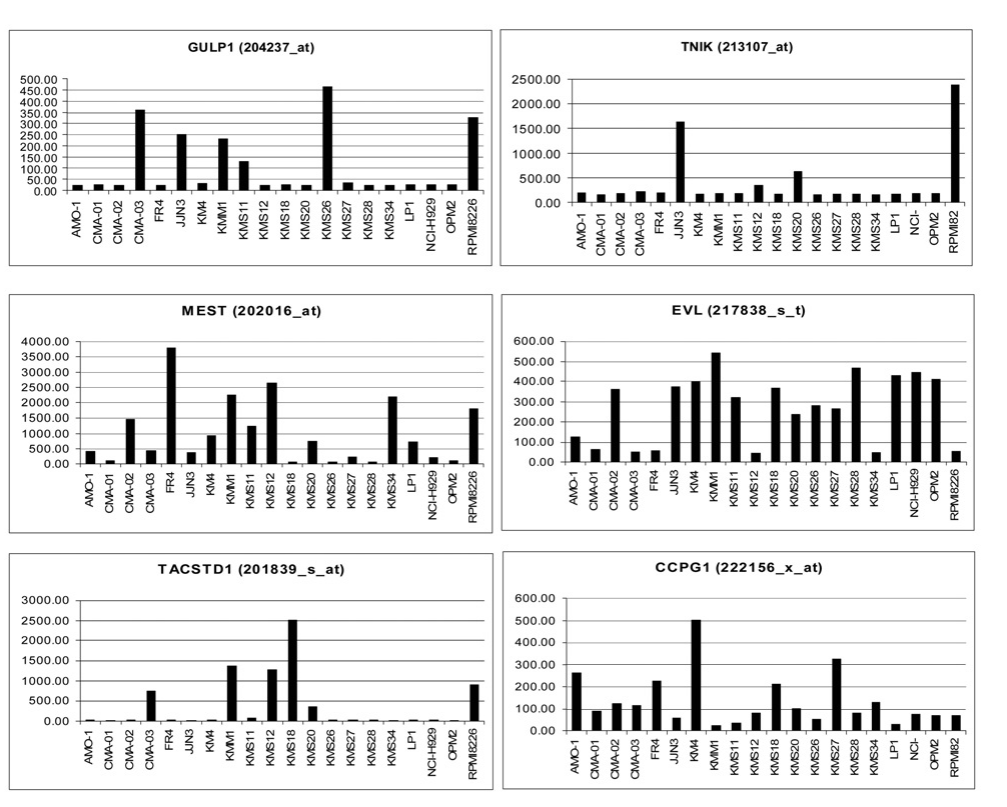
**Host gene expression levels**. Expression levels of *GULP1*, *TNIK*, *MEST*, *EVL*, *TACSTD1*, and *CCPG1 *assessed by DNA microarray analysis of HMCLs. The scaled values on the vertical axis represent the relative intensity levels as determined on HG-U133A arrays.

For each annotated probeset, we calculated the ratios of each individual expression value to the mean expression value of that probeset across the whole dataset. These ratios (or the inverse ratios whenever the individual expression value was lower than the mean) were then averaged to obtain the "average fold change" for each probeset, which is suitable as a measure of the variability of a transcript expression in a group of samples. The analysis revealed a subset of 1,032 most variable probes (specific to 799 transcripts), at an average fold change greater than 2 (2AVEFC probes). To identify which of these transcripts was a host gene for miRNA, we screened the miRNA Registry [33] (Sanger database, miRBase Sequence, version 10.0, release 2007/08/01 [34]), which includes 533 human miRNA sequences, 300 of which map to the intronic, exonic, or 3' UTR regions of 240 transcripts. Of these, 137 (specified by 261 probes) are represented on HG-U133A GeneChip array (additional file 1). By merging these 261 probes with the 1,032 2AVEFC probes from the HMCLs dataset, we identified ten 2AVEFC probes specific to six host genes, all of which contain intronic miRNA (Table 1). Specifically, we found the following pairs of host genes and intronic miRNAs: *CCPG1 *(cell cycle progression 1) and miR-628; *GULP1 *(engulfment adaptor PTB domain containing 1) and miR-561; *MEST *(mesoderm specific transcript homolog, mouse) and miR-335; *EVL *(Enah/Vasp-like) and miR-342-3p; *TACSTD1 *(tumor-associated calcium signal transducer 1) and miR-559; and *TNIK *(TRAF2 and NCK interacting kinase) and miR-569 (details in Table 1). The expression levels of the six genes are shown in Fig. 1.

**Table 1:** Selected HG-U133A probes with average fold-change higher than two in HMCLs

**HG-U133A probe**	**Host gene**	**Host gene intron**	**Intron length (bp)**	**Strand direction**	**miRNA**	**Location**	**Fold-change**
222156_x_at	*CCPG1*	intron 5	4,903	-	miR-628	15 q21	2.3
217838_s_at	*EVL*	intron 3	25,879	**+**	miR-342	14q32	2.7
204235_s_at	*GULP1*	intron 1	90,649	**+**	miR-561	2q32	4.3
204237_at	*GULP1*						4
215913_s_at	*GULP1*						2.5
202016_at	*MEST*	intron 2	1,632	**+**	miR-335	7q32	4.8
201839_s_at	*TACSTD1*	intron 5	1,874	**+**	miR-559	2p	9.7
211828_s_at	*TNIK*	intron 21	5,584	-	miR-569	3q26	3
213107_at	*TNIK*						2.4
213109_at	*TNIK*						2.3

### Expression levels of miR-335, miR-342-3p, and miR-561 correlate with those of their corresponding host genes in HMCLs

**Figure 2:**
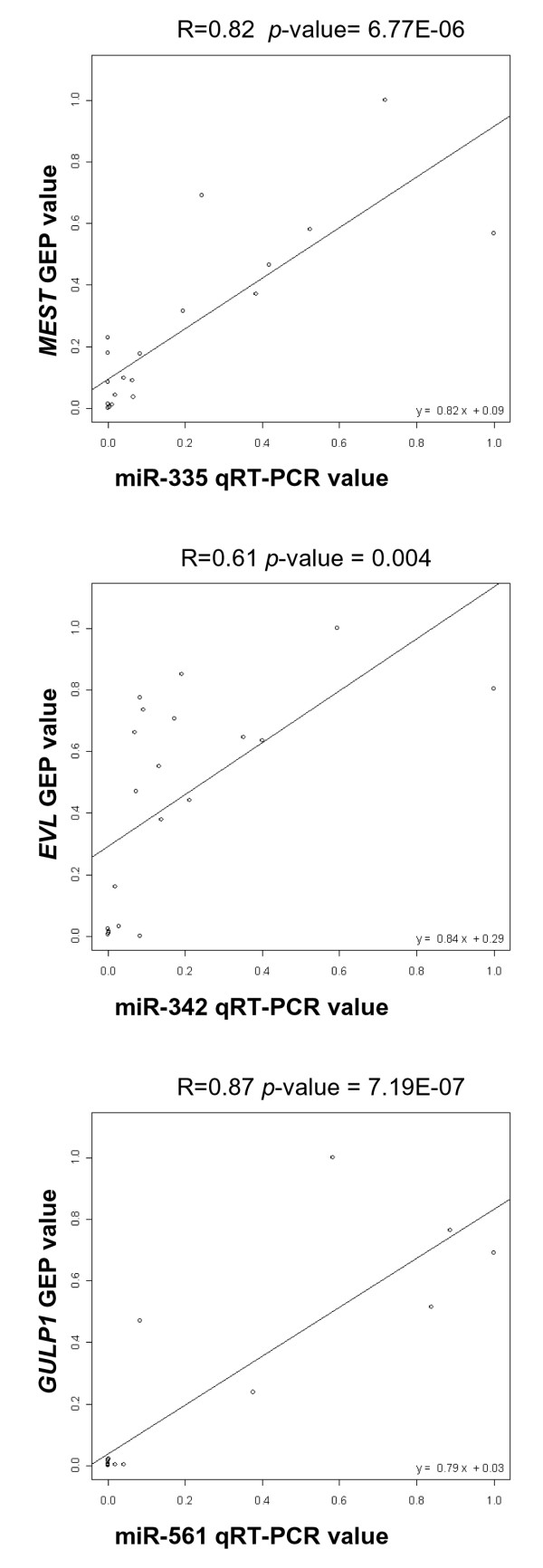
**miRNA and cognate host gene expression correlation analysis**. Correlation analyses between host genes (GEP data, ordinate) and miRNA expression levels (Q-RT-PCR, abscissa) in 20 HMCLs. To compare these data, we converted gene expression and Q-RT-PCR (expressed as 2^-ΔCt^) results between the interval values 0–1. Linear regressions, as well as the correlation coefficient R and the *p *values are indicated in each panel.

To verify whether the six intronic miRNAs were coordinately expressed with their host mRNAs in HMCLs, we investigated miRNA expression levels using quantitative-real time RT-PCR (Q-RT-PCR) assays (TaqMan^R ^microRNA assays) [35]. Although we know that the levels of mature miRNAs are not always correlated to the corresponding precursors and that the measure of pri-miRNAs or pre-miRNAs would give a more complete and reliable information about the possibly coordinated transcriptional levels of miRNAs and their host genes, we chose to evaluate only mature miRNA expression, thus focusing our interest on the biologically functional molecule. The results, normalized for the expression of the housekeeping *Z30 *small nucleolar RNA, are reported in additional file 2. Specifically, we found appreciable levels of expression for miR-335, miR-342-3p, and miR-628, whereas mir-561 and mir-559 were moderately expressed. mir-569 was barely detectable among the HMCLs in the dataset, and thus it was excluded from further analysis. A regression analysis of Q-RT-PCR miRNA values and microarray expression levels of matching host genes, conducted with R packages/software, revealed a significant correlation with the corresponding host genes for miR-335, miR-342-3p, and miR-561 (R higher than 0.6 in all cases with a *p *value < 0.005; see Fig. 2), but not for miR-559 (R = 0.12 at *p *value = 0.60) or miR-628 (R = 0.32 at *p *value = 0.15). As specified in Table 1, miR-335, miR-342-3p, miR-561, and miR-559 are all sense oriented, whereas miR-628 is in an antisense orientation with respect to its host gene.

Based on these findings, we focused our study on the miRNAs/host genes that were coordinately expressed. As described in additional file 3, the bioinformatic target prediction for miR-335, miR-342-3p, and miR-561 suggests that they might play an important role in proliferation, cell cycle control, cellular migration, and angiogenesis.

### miR-335, miR-342-3p, and miR-561 deregulations are not associated with genomic alterations

**Figure 3:**
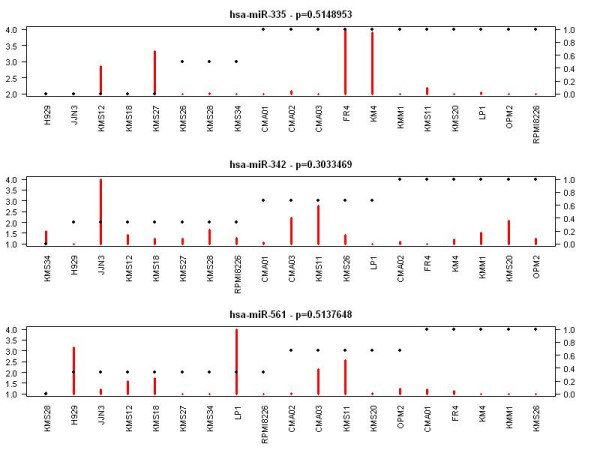
**miRNA and DNA CN correlation analysis**. Correlation analysis between DNA CN variations and miRNA expression. The red lines represent mature miRNA expression, normalized to *Z30 *small nucleolar RNA, expressed as 2^-ΔCt^, and converted between the interval values 0–1 (vertical axis on the right side). The spots represent the HMCL inferred DNA CNs (vertical axis on left side). Horizontal axis: HMCLs ordered according to increasing inferred CNs. The *p *value is indicated above each panel (Kendall's tau correlation test).

Because miRNA transcripts may be deregulated in cancer as a result of DNA CN variations [9], we investigated whether the coordinated overexpression of the three miRNAs and host transcripts was associated with CN alterations in our dataset. To this end, we performed a genome-wide DNA profiling analysis on the entire panel of HMCLs using high-density 250K SNP-arrays (Affymetrix). By referring to the University of California Santa Cruz (UCSC) Genome Browser Database [36] annotations, we positioned *MEST *between telomeric SNP_A-1960494 and centromeric SNP_A-2263405, *EVL *between telomeric SNP_A-1927639 and centromeric SNP_A-2289968, and *GULP1 *between telomeric SNP_A-4201038 and centromeric SNP_A-1941986. The CNs associated with these SNP intervals were calculated using the DNAcopy Bioconductor package (see Methods). For each HMCL, the inferred CN values and the miRNA Q-RT-PCR expression data are reported in Fig. 3. Different CNs were not reflected by a corresponding modulation of miRNA expression levels.

### miR-335, miR-342-3p, and miR-561 are overexpressed in primary MM tumors

**Figure 4:**
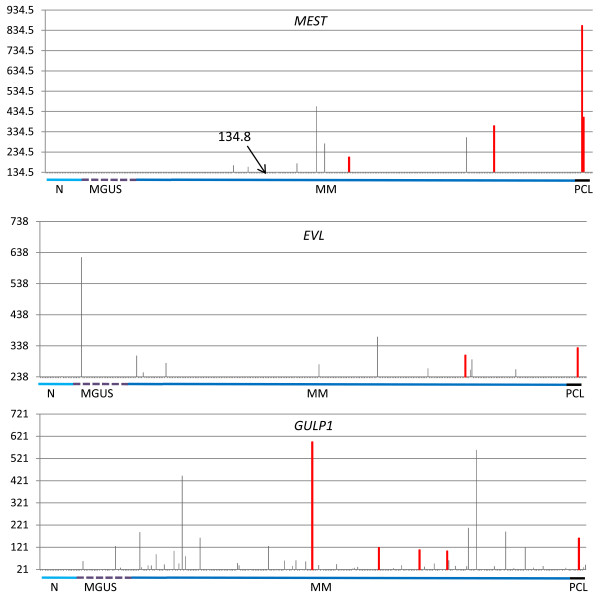
**Host gene expression level in primary tumors database**. Expression levels of *MEST*, *EVL*, and *GULP1*, as assessed by DNA microarray analysis of a patient database including samples from 24 normal donors (N), 34 MGUS, 269 MM, and nine PCL. The scaled values on the vertical axis represent the fluorescence intensity of streptavidin-PE-stained biotinylated cRNA hybridized to specific probes set on HG-U133A arrays; the baseline was set at the cut-off value calculated as mean + 3STDEV in 24 normal samples. The samples are ordered and grouped on the horizontal axis. Samples above the cut-off level and analysed by Q-RT-PCR are coloured in red; for *MEST*, the gene expression value is also reported of the sample barely exceeding the cut-off and not analysed by Q-RT-PCR.

We investigated the expression levels of the three miRNAs/host genes in normal plasma cells and MM primary tumors by querying a proprietary gene-expression-profiling (GEP) database including four normal samples, 12 monoclonal gammopathies of undetermined significance (MGUS), 132 MM, and nine plasma cell leukemias (PCLs), all profiled on HG-U133A. The MM cohort of patients was characterized for the presence of the main *IGH *chromosomal translocation, chromosome 13q deletion, 1q gain/amplification, and hyperdiploid status by fluorescence *in situ *hybridization (FISH) analyses, and stratified into the five molecular groups according to the proposed translocation/cyclin D expression (TC) classification [37]. In addition, we included in the analyses 20 normal samples, 22 MGUS, and 137 MM from two publicly available MM gene expression datasets [1,27]. To identify samples showing correlated host gene/miRNA deregulation, we considered the entire database to establish a cut-off expression level for *MEST*, *EVL*, and *GULP1 *genes by calculating the mean expression value + three standard deviations (STDEV) derived from the 24 normal samples. In particular, we found nine MM and two PCL samples with *MEST *gene expression levels exceeding the cut-off value ("positive" patients); likewise, we identified four MGUS, 47 MM, and three PCL "positive" patients for *GULP1*, and one MGUS, ten MM, and one PCL for *EVL*, although for each of these three genes some of the samples displayed a gene expression level slightly above the cut-off value (Fig. 4). The distribution of the spiked expression of *MEST*, *EVL*, and *GULP1 *with respect to the major genetic characteristics of the 132 MM patients included in the proprietary dataset are specified in additional file 4. No significant associations were found between the subgroups of MM patients deregulating *EVL *or *GULP1 *and any of the genetic aberrations that occur frequently in MM. Concerning *MEST*, the limited number of positive samples precluded contingency analysis.

To verify the correlated miRNA/host gene expression in primary tumors, we used Q-RT-PCR to test for specific miRNA expression in all of the available "positive" samples belonging to the proprietary database (Fig. 4). For each gene, a representative number of "negative" patients were also investigated (see results in additional file 5). Notably, although we tested only a limited number of samples, we found a significant correlation in expression with the corresponding host genes for miR-335, miR-342-3p, and miR-561 (R = 0.95 at *p *value = 5.03E-09, R = 0.7 at *p *value = 5E-03, and R = 0.78 at *p *value = 2E-04, respectively).

### Transcriptional profile associated with miR-335, miR-342-3p, and miR-561 overexpression

To gain insight into the possible role of these three miRNAs deregulation in MM, we looked for a specific gene expression signature associated with MM patients displaying deregulated host gene/miRNA. For each host gene, we performed a supervised analysis grouping the 269 MM samples according to the specific cut-off expression values evaluated with normal plasma cells. We identified genes that were expressed differentially among the two classes using Significant Analysis of Microarrays software (SAM). Interestingly, in the nine multiple myeloma patients overexpressing *MEST*, 70 genes were significantly upregulated. Of these, 12 are involved in the cell cycle (*p *< 0.001, DAVID tool 2006), particularly in the M phase of the cell cycle, and nine others are involved in actin polymerization and microtubule-based processes. With regard to miR-342-3p, solely the *EVL *itself resulted significantly upregulated in the ten samples overexpressing *EVL*. Finally, the 47 patients overexpressing *GULP1 *upregulated 35 probes specific to 29 genes with miscellaneous biological function. Overall the three supervised analyses did not identify downregulated transcripts in patient groups that overexpressed each miRNA; thus, no information was provided regarding putative direct targets regulated at the transcriptional level by the miRNAs themselves in MM. Full details of the differentially expressed genes resulting from the three supervised analyses are given in additional file 6.

## Discussion

Information concerning miRNA expression and deregulation in MM is still lacking. Based on the hypothesis that intronic miRNAs are coordinately expressed with host transcripts [11,14,38], we sought to identify miRNAs potentially deregulated in MM by focusing on those mapping within the intronic regions of host genes that were significantly differentially expressed in a representative panel of HMCLs profiled with U133A gene expression chips.

Following this approach, we identified six genes containing intronic miRNAs; all but one showed appreciable expression levels. For three miRNAs, miR-335, miR-342-3p, and miR-561, we demonstrated coordinated expression with their cognate protein-coding genes *MEST*, *EVL*, and *GULP1*; conversely, we did not find correlated expression of miR-628 and miR-559 and their host genes *CCPG1 *and *TACSTD1*. Notably, miR-335, miR-342-3p, miR-561, and miR-559, but not miR-628, are sense oriented with respect to the corresponding host gene. This finding is in agreement with the evidence that intronic miRNAs are usually oriented in the same direction as the pre-mRNA, and thus could be under the control of the same regulatory motifs as their host genes and processed from the same primary mRNA transcripts regulated by PolII. On the other hand, our data may also support the previous suggestion that even miRNAs in a sense orientation to annotated genes (e.g., miR-559) may have their own regulatory motifs that can be regulated by either PolII or PolIII [12].

The coregulation of these three miRNA/host gene pairs was also found in primary MM tumors. Specifically, our data showed that miR-335, miR-342-3p, and miR-561 were overexpressed in a fraction of the pathological samples with respect to normal plasma cells, without correlation to any of the known genetic alterations frequently found in MM; this finding may provide further evidence of the genetic complexity of this disease. In addition, despite the fact that miR-335 deregulation in melanoma and ovarian carcinoma was reported to be concordant with CN gain [39], neither miR-335 nor miR-342-3p and miR-561 expression levels were significantly correlated with their corresponding locus CN in our panel of HMCLs tested by SNPs arrays. This finding indicates that DNA CN alterations may not be a critical factor affecting expression of these miRNA/host genes in MM, and suggest the occurrence of epigenetic mechanisms.

Although determining the precise contribution of each miRNA to myelomagenesis was beyond the scope of this investigation, some evidence supports the potential involvement of deregulated miRNAs/host genes in myeloma. Interestingly, miR-335 and miR-342-3p were recently reported to be involved in human cancer; depleted expression of miR-335 was found to be associated with metastatic processes in human breast cancer. Specifically, miR-335 was shown to suppress metastasis by altering cell morphology and decreasing cell motility, which would limit metastatic migration [40]. Notably, we found that the fraction of primary myeloma samples overexpressing miR-335/*MEST *also showed upregulation of genes implicated in actin polymerization and microtubule-based processes. In agreement with these data, bioinformatic tools predicted that a set of genes involved in actin cytoskeleton organization and biogenesis (*DAAM1*, *ARPC5L*, *JAG1*, *MAP2*, and *RASA1*) were putative miR-335 targets (additional file 6). Therefore, one can speculate that miR-335 may be involved in MM in the physiological mechanisms reported to be altered in breast cancer, possibly influencing different processes such as plasma cell homing into the bone marrow and/or interactions with the bone marrow microenvironment. Notably, MM patients overexpressing miR-335/*MEST *also upregulated genes promoting cell proliferation, a finding apparently in contradiction with the function of miR-335 as an apoptosis permissive factor and cell cycle suppressor, as demonstrated in cortical neurosphere cultures [41]. One possible explanation might be that the cellular context and cooperation among multiple miRNAs play a key role in the final biological effect of miRNA. Alternatively, one cannot exclude a specific effect of *MEST *overexpression itself in promoting cell proliferation.

With regard to miR-342-3p, it is usually expressed in a variety of human tissues, together with its host gene *EVL*. Notably, it is specifically silenced in the majority of colorectal cancers following methylation of CpG islands located upstream of *EVL*, although the functional consequences of its silencing in carcinogenesis remain to be elucidated [42]. In addition, miR-342-3p was substantially downregulated in patients with primary myelofibrosis, polycythemia vera, or essential thrombocythemia [43], and specifically upregulated in acute promyelocytic leukemia cell lines during retinoic acid-induced differentiation [44]. Intriguingly, EVL is an actin-associated protein that is involved in a variety of processes related to cytoskeleton remodeling and cell polarity [45]; among miR-342-3p predicted targets, we recognized genes involved in actin cytoskeleton organization and biogenesis (*FHL3 *and, again, *RASA1*). One can speculate that miR-342-3p and *EVL *deregulation may target plasma cell homing into the bone marrow and/or interactions with the bone marrow microenvironment, much the same as for miR-335.

Finally, there is no information concerning the possible role of deregulated miR-561 and its cognate host gene *GULP1 *(which codes for an evolutionarily conserved adaptor protein required for efficient engulfment of apoptotic cells by phagocytes [46]) in normal or tumor cells. Because of the high frequency of *GULP1 *overexpression in MM (34%) compared with normal plasma cells, both miR-561 and its cognate host gene warrant further investigation.

## Conclusion

Our data extend the current view of miRNA origins, provide further support for the hypothesis that intronic miRNAs and their host genes may be regulated dependently, and may contribute to the understanding of their biological role in cancer. In addition, to the best of our knowledge, this is the first evidence of putative deregulated miRNAs in MM and may lead the way to identifying new biomarkers and altered molecular pathways associated with the disease.

## Competing interests

The authors declare that they have no competing interests.

## Authors' contributions

DR conceived of the study and drafted the manuscript. ML performed the quantitative RT-PCR. LM performed the genome-wide DNA profiling analysis. LA participated in the genome-wide DNA profiling analysis. AA performed the statistical analysis. SF participated in the statistical analysis. GLD helped to draft the manuscript. AN participated in study design and coordination and helped to draft the manuscript. All authors read and approved the final manuscript.

## Pre-publication history

The pre-publication history for this paper can be accessed here:



## Supplementary Material

Additional file 1 **240 host transcripts containing microRNAs**. List of all transcripts containing intronic/exonic/3' UTR miRNA or cluster of miRNA, based on the Sanger database, miRBase Sequence, version 10.0. Click here for file

Additional file 2 **MiRNA expression values (expressed as 2^-ΔCt^) by Q-RT-PCR analysis**. Expression values of miRNAs 335, 342-3p, 559, 561, 569, and 628 evaluated by Q-RT-PCR (TaqMan^R ^MicroRNA assay) in HMCLs. Click here for file

Additional file 3 **miRNA target bioinformatic prediction**. List of the putative target genes of miRNAs 335, 342-3p, and 561, predicted by merging the results from the specified prediction algorithms. The last column reports the molecular function and biological process related to each putative miRNA target as described by DAVID Tool 2006 and NetAffx. Click here for file

Additional file 4 **Distribution of the spiked expression of *MEST*, *EVL*, and *GULP1 *with respect to patients' genetic characteristics**. The main genetic characteristics of the 132 MM patients of the proprietary GEP database. The distribution of MM samples in which one of the three host genes were deregulated (according to the cut-off evaluated on normal plasma cells) with respect to genetic abnormalities is also specified. Click here for file

Additional file 5 **Host genes and miRNA expression values in primary tumors**. Expression values of host genes *MEST*, *EVL*, and *GULP1 *(GEP data expressed as relative cRNA intensity level) and corresponding miRNAs 335, 342-3p, and 561 (Q-RT-PCR data expressed as 2^-ΔCt^) in the analysed primary tumors. Click here for file

Additional file 6 **Supervised analyses of MM patients with deregulated host genes/miRNAs versus MM samples with host gene/miRNA normal expression levels**. The tables report all of the probes resulting from SAM analyses comparing MM patients overexpressing *MEST *(table 6a), *EVL *(table 6b), or *GULP1 *(table 6c) with respect to MM patients whose host gene expression levels were comparable with those of normal plasma cells. For each probe, the corresponding gene, chromosome location, involved pathway, and biological process (annotations from NetAffx), as well as the score and fold-change are specified. Click here for file
