# Supplementary material for: An integrative genomic approach reveals coordinated expression of intronic miR-335, miR-342, and miR-561 with deregulated host genes in multiple myeloma
Source: BMC Med Genomics. 2008 Aug 13;1:37. doi: 10.1186/1755-8794-1-37 (PMC2531129; doi:10.1186/1755-8794-1-37)
Supplement: Additional file 1 — 240 host transcripts containing microRNAs. List of all transcripts containing intronic/exonic/3' UTR miRNA or cluster of miRNA, based on the Sanger database, miRBase Sequence, version 10.0. [file 1755-8794-1-37-S1.pdf]

Additional file 1. 240 host genes containing intronic microRNA.

TRANSCRIPT	Intronic/exonic/3'UTR microRNA						
C22:RP4-695O20__B.9	hsa-let-7a-3	hsa-let-7b					
C21orf34	hsa-let-7c	hsa-mir-99a	hsa-mir-125b-2				
RP11-2B6.1	hsa-let-7d						
HUWE1	hsa-let-7f-2	hsa-mir-98					
WDR82	hsa-let-7g						
RCL1	hsa-mir-101-2						
PANK3	hsa-mir-103-1						
PANK2	hsa-mir-103-2						
GABRA3	hsa-mir-105-1	hsa-mir-767	hsa-mir-105-2				
PANK1	hsa-mir-107						
MIB1	hsa-mir-1-2	hsa-mir-133a-1					
RP5-963E22.4	hsa-mir-124-3						
EGFL7	hsa-mir-126						
RTL1	hsa-mir-127	hsa-mir-431	hsa-mir-433				
R3HDR1	hsa-mir-128a						
ARPP-21	hsa-mir-128b						
C20orf166	hsa-mir-133a-2	hsa-mir-1-1					
RP11-771D21.2	hsa-mir-133b						
STAB1	hsa-mir-135a-1						
RMST	hsa-mir-135a-2						
LEMD1	hsa-mir-135b						
RP11-490G2.1	hsa-mir-137						
PDE2A	hsa-mir-139						
WWP2	hsa-mir-140						
NMES1	hsa-mir-147b						
COPZ1	hsa-mir-148b						
GPC1	hsa-mir-149						
Q6ZNZ9	hsa-mir-150						
PTK2	hsa-mir-151						
COPZ2	hsa-mir-152						
PTPRN	hsa-mir-153-1						
PTPRN2	hsa-mir-153-2	hsa-mir-595					
BIC	hsa-mir-155						
DLEU2	hsa-mir-15a	hsa-mir-16-1					
SMC4	hsa-mir-16-2	hsa-mir-15b					
C13orf25	hsa-mir-17	hsa-mir-18a	hsa-mir-19a	hsa-mir-19b-1	hsa-mir-20a	hsa-mir-92a-1	
NR6A1	hsa-mir-181a-2	hsa-mir-181b-2					
RP11-31E23.1	hsa-mir-181b-1	hsa-mir-181a-1					
c22orf25	hsa-mir-185						
ZRANB2	hsa-mir-186						
CLCN5	hsa-mir-188	hsa-mir-362	hsa-mir-500	hsa-mir-501	hsa-mir-502	hsa-mir-532	hsa-mir-660
TLN2	hsa-mir-190						
DALRD3	hsa-mir-191	hsa-mir-425					
LOC388214	hsa-mir-193b	hsa-mir-365-1					
AP001187.9	hsa-mir-194-2						
AC027763.1	hsa-mir-195	hsa-mir-497					
HOXB7	hsa-mir-196a-1						
HOXC	hsa-mir-196a-2						

HOXA9	hsa-mir-196b							
FSTL1	hsa-mir-198							
DYN2	hsa-mir-199a-1	hsa-mir-638						
DYN3	hsa-mir-199a-2	hsa-mir-214						
DYN1	hsa-mir-199b							
RP13-49I15.3	hsa-mir-202							
TRPM3	hsa-mir-204							
Q7Z5Z9	hsa-mir-205							
MYH6	hsa-mir-208							
MYH7	hsa-mir-208b							
TMEM49	hsa-mir-21							
TRPM1	hsa-mir-211							
IARS2	hsa-mir-215	hsa-mir-194-1						
SLIT2	hsa-mir-218-1							
SLIT3	hsa-mir-218-2	hsa-mir-585						
NP_116284.2	hsa-mir-22							
RP5-1189B24.4	hsa-mir-220							
TBB4	hsa-mir-220b							
RL18 ST2B1 SULT2B1	hsa-mir-220c							
GABRE	hsa-mir-224	hsa-mir-452						
C9orf3	hsa-mir-24-1	hsa-mir-23b	hsa-mir-27b					
JGI_8282	hsa-mir-24-2							
MCM7	hsa-mir-25	hsa-mir-93	hsa-mir-106b					
CTDSPL	hsa-mir-26a-1							
CTDSP2	hsa-mir-26a-2							
CTDSP1	hsa-mir-26b							
LPP	hsa-mir-28							
FAM33A	hsa-mir-301a	hsa-mir-454						
LARP7	hsa-mir-302b	hsa-mir-302c	hsa-mir-302d	hsa-mir-367	hsa-mir-302a			
C6orf155	hsa-mir-30a	hsa-mir-30c-2						
NFYC	hsa-mir-30c-1	hsa-mir-30e						
RP11-354P17.9	hsa-mir-31							
C9orf5	hsa-mir-32							
ACADVL	hsa-mir-324							
ARRB1	hsa-mir-326							
ELMO3	hsa-mir-328							
EMAL2	hsa-mir-330							
MEST	hsa-mir-335							
LMTK1	hsa-mir-338	hsa-mir-657						
MGC11257	hsa-mir-339							
SREBF2	hsa-mir-33a							
SREBF1	hsa-mir-33b							
GOLI	hsa-mir-340							
EVL	hsa-mir-342							
GRID1	hsa-mir-346							
RAE1	hsa-mir-361							
AC004386.1	hsa-mir-374a	hsa-mir-545						
PRGC2 PPARGC1B	hsa-mir-378							
GC:AL132709.4	hsa-mir-381	hsa-mir-539	hsa-mir-544	hsa-mir-487b	hsa-mir-655	hsa-mir-655	hsa-mir-889	
SGCZ	hsa-mir-383							
CCDC55	hsa-mir-423							
AC004383.4	hsa-mir-424	hsa-mir-503						

HTR2C	hsa-mir-448						
CDC20B	hsa-mir-449a	hsa-mir-449b					
COL27A1	hsa-mir-455						
IGF2	hsa-mir-483						
NDE1	hsa-mir-484						
ANK1	hsa-mir-486						
ASTNX	hsa-mir-488						
CHRM2	hsa-mir-490						
KIAA1797	hsa-mir-491						
LOC160313	hsa-mir-492						
MYH7B	hsa-mir-499						
FGF13	hsa-mir-504						
ATP11C	hsa-mir-505						
MRC1L1	hsa-mir-511-1						
MRC1	hsa-mir-511-2						
ENST00000365743	hsa-mir-519a-2						
C6orf60	hsa-mir-548b						
RASF3	hsa-mir-548c						
ATAD2	hsa-mir-548d-1						
PITPNC1	hsa-mir-548d-2						
KIAA1199	hsa-mir-549						
ZNRF2	hsa-mir-550-1						
Q8N6Z3	hsa-mir-550-2						
MEGF6	hsa-mir-551a						
RTC1	hsa-mir-553						
TUFT1	hsa-mir-554						
ASH1L	hsa-mir-555						
NOS1AP	hsa-mir-556						
BIRC6	hsa-mir-558						
TACSTD1	hsa-mir-559						
CU099	hsa-mir-560						
GULP1	hsa-mir-561						
Q8N9K9	hsa-mir-562						
TMM42	hsa-mir-564						
SEMA3F	hsa-mir-566						
c3orf52	hsa-mir-567						
TNIK	hsa-mir-569						
bcm2738	hsa-mir-570						
ZN141	hsa-mir-571						
RNPS1	hsa-mir-572						
NXP20	hsa-mir-574						
SCD5	hsa-mir-575						
SC24B	hsa-mir-576						
UGT8	hsa-mir-577						
CBPE	hsa-mir-578						
ZFR	hsa-mir-579						
LMBRD2	hsa-mir-580						
ARL15	hsa-mir-581						
PDE4D	hsa-mir-582						
S3TC2	hsa-mir-584						
SUPT3H	hsa-mir-586						
RP1-60O19.1	hsa-mir-587						
FBXL18	hsa-mir-589						

IF4H	hsa-mir-590						
SLC25A13	hsa-mir-591						
GRM8	hsa-mir-592						
SND1	hsa-mir-593						
TNKS	hsa-mir-597						
XKR6	hsa-mir-598						
VPS13B	hsa-mir-599	hsa-mir-875					
STRBP	hsa-mir-600						
DENND1A	hsa-mir-601						
EHMT1	hsa-mir-602						
Q5T5P3	hsa-mir-603						
SVIL	hsa-mir-604	hsa-mir-938					
PRKG1	hsa-mir-605						
C10orf11	hsa-mir-606						
SEMA4G	hsa-mir-608						
c10orf79	hsa-mir-609						
KI18A	hsa-mir-610						
CK010	hsa-mir-611						
APLD1	hsa-mir-613						
bcm3978	hsa-mir-614						
HOXC5	hsa-mir-615						
DDIT3	hsa-mir-616						
LIN7A	hsa-mir-617	hsa-mir-618					
SSH1	hsa-mir-619						
THRAP2	hsa-mir-620						
SLC25A15	hsa-mir-621						
CMTM8	hsa-mir-622						
PHGDHL1	hsa-mir-623						
STRN3	hsa-mir-624						
FUT8	hsa-mir-625						
Q8IWI9	hsa-mir-626						
VPS39	hsa-mir-627						
CCPG1	hsa-mir-628						
TLE3	hsa-mir-629						
ARIH1	hsa-mir-630						
NEIL1	hsa-mir-631						
ZNF207	hsa-mir-632						
KPCA	hsa-mir-634						
WIPI1	hsa-mir-635						
SFRS2	hsa-mir-636						
DAPK3	hsa-mir-637						
GPSN2	hsa-mir-639						
P66A	hsa-mir-640						
AKT2	hsa-mir-641						
GIPR	hsa-mir-642						
ZN766	hsa-mir-643						
ITCH	hsa-mir-644						
RP5-1043L13.1	hsa-mir-646						
UCKL1	hsa-mir-647						
TM164	hsa-mir-652						
CALCR	hsa-mir-653	hsa-mir-489					
ANR54	hsa-mir-658						
PLEC1	hsa-mir-661						

LOC401827	hsa-mir-662						
RP3-410C9.1	hsa-mir-663						
CHGUT	hsa-mir-671						
H19	hsa-mir-675						
HNRPK	hsa-mir-7-1						
c19orf30	hsa-mir-7-3						
MP2K4	hsa-mir-744						
BCAR3	hsa-mir-760						
ARHGEF11	hsa-mir-765						
SEPT6	hsa-mir-766						
AP1G1	hsa-mir-768						
MEG3	hsa-mir-770						
RUNX1	hsa-mir-802						
KLHL3	hsa-mir-874						
DDR1	hsa-mir-877						
ABCF1	hsa-mir-877						
ATP2B2	hsa-mir-885						
C1orf61	hsa-mir-9-1						
SOX5	hsa-mir-920						
FA78B	hsa-mir-921						
KIAA0226	hsa-mir-922						
UN45B	hsa-mir-923						
HsG9510	hsa-mir-9-3						
ATF2	hsa-mir-933						
VGLL1	hsa-mir-934						
CCG8	hsa-mir-935						
COL17A1	hsa-mir-936						
LAP4	hsa-mir-937						
CPSF1	hsa-mir-939						
DNJC5	hsa-mir-941-1	hsa-mir-941-2	hsa-mir-941-3				
TTF2	hsa-mir-942						
NELFA	hsa-mir-943						
P73L	hsa-mir-944						
ABLM2	hsa-mir-95						
