# Supplementary material for: An integrative genomic approach reveals coordinated expression of intronic miR-335, miR-342, and miR-561 with deregulated host genes in multiple myeloma
Source: BMC Med Genomics. 2008 Aug 13;1:37. doi: 10.1186/1755-8794-1-37 (PMC2531129; doi:10.1186/1755-8794-1-37)
Supplement: Additional file 2 — MiRNA expression values (expressed as 2-ΔCt) by Q-RT-PCR analysis. Expression values of miRNAs 335, 342-3p, 559, 561, 569, and 628 evaluated by Q-RT-PCR (TaqManR MicroRNA assay) in HMCLs. [file 1755-8794-1-37-S2.pdf]

Additional file 2. MiRNA expression values (expressed as $2^{-\Delta Ct}$) by qRT-PCR analysis.

HMCLs	**335**	**342**	**559**	**561**	**569**	**628**
AMO-1	9.28E-02	1.55E-01	2.10E-05	1.76E-08	8.08E-09	4.43E-02
CMA-01	1.75E-02	2.35E-01	8.49E-01	1.26E-04	5.39E-08	1.53E-01
CMA-02	5.51E-01	3.31E+00	2.18E-07	1.80E-09	1.88E-08	9.08E-02
CMA-03	6.01E-02	1.88E-02	3.13E-05	2.66E-03	1.06E-08	2.63E-02
FR4	1.03E+00	1.72E-03	3.18E-08	1.47E-06	5.96E-08	3.79E-01
H929	9.58E-02	8.25E+00	5.69E-07	2.69E-09	2.97E-08	6.14E-02
JJN3	7.98E-05	5.80E-01	3.42E-05	2.52E-03	7.86E-06	1.33E-02
KM4	3.40E-05	1.43E+00	2.28E-09	2.28E-09	2.69E-09	1.48E-02
KMM1	7.52E-01	4.91E+00	3.88E-07	3.01E-08	4.69E-09	1.53E-02
KMS11	2.80E-01	1.10E+00	2.07E-04	1.13E-03	3.79E-09	7.04E-03
KMS12	3.50E-01	6.91E-01	1.42E-08	1.42E-08	2.18E-09	2.92E-02
KMS18	7.00E-03	2.91E+00	9.76E-08	1.17E-08	8.57E-08	2.83E-02
KMS20	3.46E-05	1.15E+00	1.96E-08	1.96E-08	1.57E-09	8.19E-03
KMS26	1.16E-03	6.08E-01	9.93E-06	1.75E-03	1.87E-07	2.76E-03
KMS27	2.84E-02	1.75E+00	7.64E-07	1.36E-09	1.18E-09	2.15E-01
KMS28 BM	5.64E-05	1.58E+00	7.67E-10	4.35E-08	1.48E-09	1.05E-02
KMS34	1.43E+00	1.37E-02	2.60E-09	5.93E-05	1.13E-07	3.64E-02
LP1	1.18E-01	6.85E-01	2.31E-05	2.49E-04	2.79E-09	8.69E-03
OPM2	3.01E-08	7.66E-01	2.30E-09	1.00E-05	5.85E-07	8.42E-03
RPMI-8226	5.98E-01	2.79E-02	2.43E-04	3.00E-03	4.56E-09	2.95E-02
