# Supplementary material for: An integrative genomic approach reveals coordinated expression of intronic miR-335, miR-342, and miR-561 with deregulated host genes in multiple myeloma
Source: BMC Med Genomics. 2008 Aug 13;1:37. doi: 10.1186/1755-8794-1-37 (PMC2531129; doi:10.1186/1755-8794-1-37)
Supplement: Additional file 4 — Distribution of the spiked expression of MEST, EVL, and GULP1 with respect to patients' genetic characteristics. The main genetic characteristics of the 132 MM patients of the proprietary GEP database. The distribution of MM samples in which one of the three host genes were deregulated (according to the cut-off evaluated on normal plasma cells) with respect to genetic abnormalities is also specified. [file 1755-8794-1-37-S4.pdf]

Additional file 4. Distribution of the spiked expression of *MEST, EVL,* and *GULP1* genes with respect to patients' genetic characteristics

Variables	Total (n=132)	Normal *MEST* (N=128)	OE* *MEST* (N=4)	Normal *EVL* (N=125)	OE* *EVL* (N=7)	Normal *GULP1* (N=109)	OE* *GULP1* (N=23)
TC1 t(11;14) or t(6;14)	29						
no		100	3	98	5	84	19
yes		28	1	27	2	25	4
TC2	24						
no		104	4	102	6	88	20
yes		24	0	23	1	21	3
TC3	49						
no		81	2	78	5	72	11
yes		47	2	47	2	37	12
TC4 t(4;14)	24						
no		105	3	103	5	86	22
yes		23	1	22	2	23	1
TC5 t(14;16) or t(14;20)	6						
no		122	4	119	7	106	20
yes		6	0	6	0	3	3
HD†	43						
no		53	2	52	3	49	8
yes		41	2	40	3	49	13
missing		34	0	33	1	15	2
del13‡	61						
no		56	1	55	2	49	8
yes		58	3	56	5	49	13
missing		14	0	14	0	15	2
1q gain	54						
no		51	1	49	3	47	5
yes		51	3	50	4	42	12
missing		26	0	26	0	20	6

*OE= overexpressed, †HD= hyperdiploid status, ‡del13= chromosome 13q deletion.
