# Supplementary material for: An integrative genomic approach reveals coordinated expression of intronic miR-335, miR-342, and miR-561 with deregulated host genes in multiple myeloma
Source: BMC Med Genomics. 2008 Aug 13;1:37. doi: 10.1186/1755-8794-1-37 (PMC2531129; doi:10.1186/1755-8794-1-37)
Supplement: Additional file 5 — Host genes and miRNA expression values in primary tumors. Expression values of host genes MEST, EVL, and GULP1 (GEP data expressed as relative cRNA intensity level) and corresponding miRNAs 335, 342-3p, and 561 (Q-RT-PCR data expressed as 2-ΔCt) in the analysed primary tumors. [file 1755-8794-1-37-S5.pdf]

Additional file 5. Host genes (GEP data expressed as relative cRNA intensity level) and miRNA (qRT-PCR data expressed as $2^{-\Delta Ct}$) expression values in primary tumors.

**PRIMARY TUMORS**	***MEST***	**335**	***EVL***	**342**	***GULP1***	**561**
MM-004	44.6	1.18E-02	nd	nd	**594.3**	**3.65E-04**
MM-015	42.5	1.47E-02	63.5	1.02E-01	13.4	1.79E-09
MM-016	125.3	1.22E-02	46.3	1.40E-03	13.9	2.51E-10
MM-030	75.2	4.64E-02	63.7	9.84E-02	12.7	8.57E-10
MM-031	nd	nd	40.9	9.28E-03	nd	nd
MM-038	53.0	1.35E-02	133.2	7.06E-02	13.8	3.89E-08
MM-040	**208.5**	**1.17E-01**	nd	nd	13.6	2.98E-09
MM-042	65.5	5.26E-02	74.8	2.20E-01	17.1	1.38E-09
MM-043	44.0	4.84E-03	123.7	1.41E-01	13.2	1.81E-06
MM-049	113.7	2.99E-02	104.7	2.90E-02	25.1	1.43E-06
MM-078	35.6	4.19E-03	nd	nd	**118.8**	**1.06E-04**
MM-123	101.7	5.56E-02	nd	nd	**108.5**	**2.97E-06**
MM-154	34.9	5.54E-03	64.8	4.30E-02	**103.2**	**1.85E-05**
MM-177	39.5	2.29E-02	71.0	6.94E-02	14.6	2.94E-09
MM-179	nd	nd	**307.3**	**1.27E-01**	nd	nd
MM-206	**362.6**	**1.06E-01**	104.0	3.48E-01	12.2	6.26E-08
PCL-004	**857.4**	**2.44E-01**	nd	nd	13.0	2.31E-09
PCL-005	**405.9**	**1.51E-01**	**331.1**	**2.58E+00**	13.9	4.43E-09
PCL-006	52.6	6.26E-03	34.8	3.95E-02	**161.0**	**4.19E-04**

Samples with host gene expression value above the cut off are marked in bold.
