# Supplementary material for: An integrative genomic approach reveals coordinated expression of intronic miR-335, miR-342, and miR-561 with deregulated host genes in multiple myeloma
Source: BMC Med Genomics. 2008 Aug 13;1:37. doi: 10.1186/1755-8794-1-37 (PMC2531129; doi:10.1186/1755-8794-1-37)
Supplement: Additional file 6 — Supervised analyses of MM patients with deregulated host genes/miRNAs versus MM samples with host gene/miRNA normal expression levels. The tables report all of the probes resulting from SAM analyses comparing MM patients overexpressing MEST (table 6a), EVL (table 6b), or GULP1 (table 6c) with respect to MM patients whose host gene expression levels were comparable with those of normal plasma cells. For each probe, the corresponding gene, chromosome location, involved pathway, and biological process (annotations from NetAffx), as well as the score and fold-change are specified. [file 1755-8794-1-37-S6.pdf]

Additional file 6. Supervised analyses of MM patients deregulating host gene/miRNA *versus* MM samples with host gene/miRNA normal expression levels.

Table 6a. Eighty-five up-regulated probes in MMs overexpressing MEST/mir-335, ordered by gene name.

Probe ID	GENE	NAME	Chrom. Location	Pathway	biological process	Score(d)	Fold Change
39249_at	AQP3	aquaporin 3 (Gill blood group)	9p13	---	transport	7.54	2.31
203747_at	AQP3	---	---	---		6.65	1.93
218115_at	ASF1B	ASF1 anti-silencing function 1 homolog B (S. cerevisiae)	19p13.12	---	chromatin assembly or disassembly	6.43	1.79
219918_s_at	ASPM	asp (abnormal spindle) homolog, microcephaly associated (Drosophila)	1q31	---	cell cycle	6.98	3.61
218782_s_at	ATAD2	ATPase family, AAA domain containing 2	8q24.13	---	---	6.23	1.96
209464_at	AURKB	aurora kinase B	17p13.1	---	protein amino acid phosphorylation /// cell cycle	5.74	1.54
202095_s_at	BIRC5	baculoviral IAP repeat-containing 5 (survivin)	17q25	Apoptosis	G2/M transition of mitotic cell cycle /// negative regulation of caspase activity	6.32	2.01
215509_s_at	BUB1	BUB1 budding uninhibited by benzimidazoles 1 homolog (yeast)	2q14	Cell cycle	protein amino acid phosphorylation /// mitosis	7.45	1.83
209642_at	BUB1	---	---	---	---	6.01	2.53
203755_at	BUB1B	BUB1 budding uninhibited by benzimidazoles 1 homolog beta (yeast)	15q15	Cell cycle	apoptosis /// mitotic cell cycle checkpoint	6.46	2.65
213410_at	C10orf137	chromosome 10 open reading frame 137	10q26.13-q26.2	---	regulation of transcription, DNA-dependent	5.71	2.20
203418_at	CCNA2	cyclin A2	4q25-q31	Cell cycle	regulation of progression through cell cycle	8.89	1.94
213226_at	CCNA2	---	---	---	---	7.71	1.83

203214_x_at	CDC2	cell division cycle 2, G1 to S and G2 to M	10q21.1	Cell cycle	regulation of progression through cell cycle	8.83	2.19
210559_s_at	CDC2	---	---	---	---	7.68	2.64
203213_at	CDC2	---	---	---	---	6.81	2.90
202870_s_at	CDC20	cell division cycle 20 homolog (S. cerevisiae)	1p34.1	Cell cycle	regulation of progression through cell cycle	5.75	1.84
203967_at	CDC6	cell division cycle 6 homolog (S. cerevisiae)	17q21.3	Cell cycle	DNA replication checkpoint	6.16	2.11
221520_s_at	CDCA8	cell division cycle associated 8	1p34.3	---	cell cycle	6.28	1.48
204962_s_at	CENPA	centromere protein A	2p24-p21	---	nucleosome assembly	7.03	1.87
205046_at	CENPE	centromere protein E, 312kDa	4q24-q25	---	mitotic metaphase	7.31	1.63
207828_s_at	CENPF	centromere protein F, 350/400ka (mitosin)	1q32-q41	---	G2 phase of mitotic cell cycle	6.76	2.17
205393_s_at	CHEK1	CHK1 checkpoint homolog (S. pombe)	11q24-q24	Cell cycle	DNA damage checkpoint	7.13	1.71
220295_x_at	DEPDC1 /// LOC730888	DEP domain containing 1 /// similar to DEP domain containing 1	1p31.2	---	signal transduction	7.16	1.96
219990_at	E2F8	E2F transcription factor 8	11p15.1	---	regulation of progression through cell cycle	6.16	1.87
219454_at	EGFL6	EGF-like-domain, multiple 6	Xp22	---	cell cycle	5.79	1.85
38158_at	ESPL1	extra spindle pole bodies homolog 1 (S. cerevisiae)	12q	Cell cycle	mitotic sister chromatid segregation	7.76	1.69
206102_at	GINS1	GINS complex subunit 1 (Psf1 homolog)	20p11.21	---	DNA replication	8.22	2.47
220993_s_at	GPR63	G protein-coupled receptor 63	6q16.1-q16.3	GPCRDB Class A Rhodopsin-like	G-protein coupled receptor protein signaling pathway	5.68	2.05
213793_s_at	HOMER1	homer homolog 1 (Drosophila)	5q14.2	---	metabotropic glutamate receptor, phospholipase C activating pathway	5.68	3.48

205543_at	HSPA4L	heat shock 70kDa protein 4-like	4q28	---	protein folding /// response to unfolded protein	5.70	1.79
210587_at	INHBE	inhibin, beta E	12q13.3	---	---	6.16	5.68
204444_at	KIF11	kinesin family member 11	10q24.1	---	microtubule-based movement	6.14	2.39
219306_at	KIF15	kinesin family member 15	3p21.31	---	microtubule-based movement	8.30	2.10
218755_at	KIF20A	kinesin family member 20A	5q31	---	microtubule-based movement	7.62	1.68
204411_at	KIF21B	kinesin family member 21B	1pter-q31.3	---	microtubule-based movement	6.25	2.55
209408_at	KIF2C	kinesin family member 2C	1p34.1	---	microtubule-based movement	6.11	1.39
218355_at	KIF4A	kinesin family member 4A	Xq13.1	---	microtubule-based movement	5.84	1.77
211042_x_at	MCAM	melanoma cell adhesion molecule	11q23.3	---	cell adhesion	5.75	1.51
220651_s_at	MCM10	minichromosome maintenance complex component 10	10p13	---	regulation of progression through cell cycle	7.41	1.64
222037_at	MCM4	minichromosome maintenance complex component 4	8q11.2	Cell cycle	DNA replication	6.92	1.74
222036_s_at	MCM4	---	---	---	---	6.24	1.76
201930_at	MCM6	minichromosome maintenance complex component 6	2q21	Cell cycle	DNA replication	5.74	2.28
202016_at	MEST	mesoderm specific transcript homolog (mouse)	7q32	---	---	19.86	4.54
212021_s_at	MKI67	antigen identified by monoclonal antibody Ki-67	10q25-qter	---	regulation of progression through cell cycle	8.92	2.17
212020_s_at	MKI67	---	---	---	---	8.25	1.83
212022_s_at	MKI67	---	---	---	---	5.96	1.70
212789_at	NCAPD3	non-SMC condensin II complex, subunit D3	11q25	---	mitotic chromosome condensation	6.36	1.72
218663_at	NCAPG	non-SMC condensin I complex, subunit G	4p15.33	---	mitotic chromosome condensation	6.79	1.78

204641_at	NEK2	NIMA (never in mitosis gene a)-related kinase 2	1q32.2-q41	---	regulation of progression through cell cycle	7.09	2.78
218039_at	NUSAP1	nucleolar and spindle associated protein 1	15q15.1	---	mitotic sister chromatid segregation	6.26	2.96
217499_x_at	OR7E37P	olfactory receptor, family 7, subfamily E, member 37 pseudogene	13q14.11	---	---	5.72	1.55
201202_at	PCNA	proliferating cell nuclear antigen	20pter-p12	Cell cycle	regulation of progression through cell cycle	6.55	2.76
204887_s_at	PLK4	polo-like kinase 4 (Drosophila)	4q28	---	regulation of progression through cell cycle	6.78	1.53
203883_s_at	RAB11FIP2	RAB11 family interacting protein 2 (class I)	10q26.11	---	protein transport	5.69	1.96
222077_s_at	RACGAP1	Rac GTPase activating protein 1	12q13.13	S1P Signaling	cytokinesis, initiation of separation	6.97	3.39
209773_s_at	RRM2	ribonucleotide reductase M2 polypeptide	2p25-p24	---	DNA replication	6.85	4.06
201890_at	RRM2					6.17	3.95
219493_at	SHCBP1	SHC SH2-domain binding protein 1	16q11.2	---	---	6.25	3.15
203625_x_at	SKP2	S-phase kinase-associated protein 2 (p45)	5p13	Cell cycle	regulation of progression through cell cycle	8.99	2.03
206052_s_at	SLBP	stem-loop (histone) binding protein	4p16.3	---	mRNA processing	5.89	2.19
204240_s_at	SMC2	structural maintenance of chromosomes 2	9q31.1	---	mitotic chromosome condensation	5.82	2.33
208608_s_at	SNTB1	syntrophin, beta 1 (dystrophin-associated protein A1, 59kDa, basic component 1)	8q23-q24	---	muscle contraction	5.89	1.55
206626_x_at	SSX1	synovial sarcoma, X breakpoint 1	Xp11.23-p11.22	---	transcription	7.17	4.24
216471_x_at	SSX2	synovial sarcoma, X breakpoint 2	Xp11.23-p11.22	---	transcription	6.98	2.21
210497_x_at	SSX2	---	---	---	---	6.81	2.19

210394_x_at	SSX4 /// SSX4B	synovial sarcoma, X breakpoint 4 /// synovial sarcoma, X breakpoint 4B	Xp11.23	---	transcription	6.01	2.41
218308_at	TACC3	transforming, acidic coiled-coil containing protein 3	4p16.3	---	regulation of progression through cell cycle	6.02	1.63
222116_s_at	TBC1D16	TBC1 domain family, member 16	17q25.3	---	regulation of Rab GTPase activity	6.14	2.06
209753_s_at	TMPO	thymopoietin	12q22	---	regulation of transcription	6.76	1.84
201292_at	TOP2A	topoisomerase (DNA) II alpha 170kDa	17q21-q22	---	DNA metabolic process /// chromosome segregation	8.28	4.89
201291_s_at	TOP2A	---	---	---	---	6.20	3.69
210052_s_at	TPX2	TPX2, microtubule-associated, homolog (Xenopus laevis)	20q11.2	---	mitosis /// cell proliferation	9.85	2.44
204033_at	TRIP13	thyroid hormone receptor interactor 13	5p15.33	---	transcription from RNA polymerase II promoter	6.18	1.71
218156_s_at	TSR1	TSR1, 20S rRNA accumulation, homolog (S. cerevisiae)	17p13.3	---	---	6.06	1.83
212639_x_at	TUBA1B	tubulin, alpha 1b	12q13.12	---	microtubule-based process	6.05	1.93
213646_x_at	TUBA1B	---	---	---	---	5.83	1.90
201090_x_at	TUBA1B	---	---	---	---	5.82	1.93
209026_x_at	TUBB	tubulin, beta	6p21.33	Glycolysis and Gluconeogenesis	microtubule-based process	7.93	2.30
211714_x_at	TUBB	---	---	---	---	7.07	2.20
212320_at	TUBB	---	---	---	---	5.76	1.98
202589_at	TYMS	thymidylate synthetase	18p11.32	---	nucleic acid metabolic process	7.07	3.34
202954_at	UBE2C	ubiquitin-conjugating enzyme E2C	20q13.12	---	ubiquitin-dependent protein catabolic process	6.42	1.75
214061_at	WDR67	WD repeat domain 67	8q24.13	---	regulation of Rab GTPase activity	5.88	1.44

204026_s_at	ZWINT	ZW10 interactor	10q21-q22	---	mitotic sister chromatid segregation	6.52	3.20

Table 6b. One up-regulated probe in MMs overexpressing EVL/mir-342.

**Probe ID**	**GENE**	**NAME**	**Chrom. Location**	**Pathway**	**biological process**	**Score(d)**	**Fold Change**
217838_s_at	EVL	Enah/Vasp-like	14q32.2	---	actin filament organization	6.83	6.9

Table 6c. Thirty-five up-regulated probes in MMs overexpressing GULP1/mir-561, ordered by gene name.

**Probe ID**	**GENE**	**NAME**	**Chrom. Location**	**Pathway**	**biological process**	**Score(d)**	**Fold Change**
221815_at	ABHD2	abhydrolase domain containing 2	15q26.1	---	negative regulation of cell migration	4.98	1.30
63825_at	ABHD2	---	---	---	---	4.80	1.39
87100_at	ABHD2	---	---	---	---	4.43	1.53
210461_s_at	ABLIM1	actin binding LIM protein 1	10q25	---	cytoskeleton organization and biogenesis	4.44	1.14
219496_at	ANKRD57	ankyrin repeat domain 57	2q13	---	---	4.30	1.58
205068_s_at	ARHGAP26	Rho GTPase activating protein 26	5q31	---	signal transduction	4.57	1.33
219747_at	C4orf31	chromosome 4 open reading frame 31	4q27	---	---	4.11	2.03
217196_s_at	CAMSAP1L 1	calmodulin regulated spectrin-associated protein 1-like 1	1q32.1	---	---	4.78	1.48
210895_s_at	CD86	CD86 molecule	3q21	Inflammatory Response	immune response /// cell-cell signaling	4.13	1.50
201953_at	CIB1	calcium and integrin binding 1 (calmyrin)	15q25.3-q26	---	double-strand break repair	4.28	1.26
203953_s_at	CLDN3	claudin 3	7q11.23	---	calcium-independent cell-cell adhesion	5.19	1.32
203954_x_at	CLDN3	---	---	---	---	4.81	2.65
213415_at	CLIC2	chloride intracellular channel 2	Xq28	---	transport	4.52	1.63
206284_x_at	CLTB	clathrin, light chain (Lcb)	4q2-q3|5q35	---	intracellular protein transport	4.50	1.21
201200_at	CREG1	cellular repressor of E1A-stimulated genes 1	1q24	---	regulation of cell growth	5.37	1.55
206595_at	CST6	cystatin E/M	11q13	---	epidermis development	5.27	3.41

205225_at	ESR1	estrogen receptor 1	6q25.1	Nuclear Receptors	regulation of transcription, DNA-dependent	5.14	1.78
213766_x_at	GNA11	Guanine nucleotide binding protein (G protein), alpha 11 (Gq class)	19p13.3	G Protein Signaling	skeletal development	4.84	1.23
204235_s_at	GULP1	GULP, engulfment adaptor PTB domain containing 1	2q32.3-q33	---	transport /// phagocytosis	10.59	3.06
204237_at	GULP1	---	---	---	---	9.79	6.27
215913_s_at	GULP1	---	---	---	---	9.34	6.48
218450_at	HEBP1	heme binding protein 1	12p13.1	---	circadian rhythm	4.72	1.45
212687_at	LIMS1	LIM and senescent cell antigen-like domains 1	2q12.3-q13	---	cell aging	4.29	1.42
200704_at	LITAF	lipopolysaccharide-induced TNF factor	16p13.13	---	regulation of transcription, DNA-dependent	4.52	1.41
200706_s_at	LITAF	---	---	---	---	4.46	1.50
201866_s_at	NR3C1	nuclear receptor subfamily 3, group C, member 1 (glucocorticoid receptor)	5q31.3	Apoptosis_KEGG /// Nuclear_Receptors	transcription, DNA-dependent	4.12	1.34
213062_at	NTAN1	N-terminal asparagine amidase	16p13.11	---	memory /// adult locomotory behavior	4.39	1.48
205590_at	RASGRP1	RAS guanyl releasing protein 1 (calcium and DAG-regulated)	15q15	---	signal transduction	4.33	1.97
212724_at	RND3	Rho family GTPase 3	2q23.3	---	cell adhesion	4.92	3.14
219370_at	RPRM	reprimo, TP53 dependent G2 arrest mediator candidate	2q23.3	---	cell cycle arrest	4.22	1.20
212698_s_at	SEPT10	septin 10	2q13	---	cell cycle	4.22	1.59
202011_at	TJP1	tight junction protein 1 (zona occludens 1)	15q13	---	intercellular junction assembly	4.74	1.57
204529_s_at	TOX	thymocyte selection-associated high mobility group box	8q12.1	---	regulation of transcription, DNA-dependent	5.07	1.95
211982_x_at	XPO6	exportin 6	16p11.2	---	protein import into nucleus, docking	4.23	1.14
211962_s_at	ZFP36L1	zinc finger protein 36, C3H type-like 1	14q22-q24	---	regulation of mRNA stability	4.41	1.86
